# L-Cysteine Treatment Delays Leaf Senescence in Chinese Flowering Cabbage by Regulating ROS Metabolism and Stimulating Endogenous H_2_S Production

**DOI:** 10.3390/foods14010029

**Published:** 2024-12-25

**Authors:** Linzhi Gan, Zhenliang Mou, Jianye Chen, Wei Shan, Jianfei Kuang, Wangjin Lu, Yating Zhao, Wei Wei

**Affiliations:** 1 State Key Laboratory for Conservation and Utilization of Subtropical Agro-Bioresources/Guangdong Provincial Key Laboratory of Postharvest Science of Fruits and Vegetables/Engineering Research Center of Southern Horticultural Products Preservation, Ministry of Education, College of Horticulture, South China Agricultural University, Guangzhou 510642, China; linzhigan@stu.scau.edu.cn (L.G.); mouzhenliang@stu.scau.edu.cn (Z.M.); chenjianye@scau.edu.cn (J.C.); shanwei@scau.edu.cn (W.S.); jfkuang@scau.edu.cn (J.K.); wjlu@scau.edu.cn (W.L.); 2 College of Food Science and Pharmacy, Xinjiang Agricultural University, Urumqi 830052, China; zhaoyt@xjau.edu.cn

**Keywords:** Chinese flowering cabbage, leaf senescence, L-cysteine, reactive oxygen species, hydrogen sulfide

## Abstract

Leaf senescence is a major concern for postharvest leafy vegetables, as leaves are highly prone to yellowing and nutrient loss, resulting in reduced commercial value and limited shelf-life. This study aimed to investigate the effect of L-cysteine (L-cys) on postharvest Chinese flowering cabbage stored at 20 °C. The results showed that 0.5 g L^−1^ L-cys treatment effectively slowed leaf senescence by downregulating chlorophyll degradation genes *(BrNYC1, BrNOL*, *BrPPH*, *BrPAO*, *BrNYE*, and *BrSAGs*) and senescence marker gene *BrSAG12*. Moreover, this treatment exhibited positive influence on the nutritional quality of cabbage. Also, L-cys treatment maintained ROS homeostasis, preventing excessive ROS accumulation and lipid membrane oxidation. L-cys treatment also maintained a higher total antioxidant capacity and scavenging rate of •OH and O_2_^•−^. Additionally, L-cys treatment maintained high levels of ascorbate and glutathione and activated antioxidant enzymes (superoxide dismutase, peroxidase, and catalase) and the expression of the encoding genes. Furthermore, L-cys treatment elevated endogenous H_2_S levels, which are correlated with increased L-cysteine desulfhydrase activity and the upregulation of H_2_S biosynthesis-related genes. These findings suggest that L-cys can delay leaf senescence by reducing chlorophyll breakdown, maintaining ROS homeostasis, and stimulating endogenous H_2_S production.

## 1. Introduction

Chinese flowering cabbage (*Brassica rapa* ssp. *Parachinensisis*) is known throughout China for its high nutritional value and sweet flavor. However, the metabolism of the cabbage, as a leafy vegetable, is highly active after harvest, making it susceptible to nutrient loss, wilting, and leaf yellowing, which significantly reduce its shelf life and commercial value [1]. Although there are some postharvest preservation technologies for leafy vegetables, including chemical measures (melatonin [2], 6-Benzylaminopurine (6-BA) [3], and 1-methylcyclopropene (1-MCP) [4]) and physical measures (ultraviolet-C (UV-C) [5] and modified atmosphere packaging [6]), these strategies are associated with high cost, chemical residues, and lack of durability, thus impacting the sensory and nutritional properties and food safety of the vegetables. Therefore, cost-effective methods using non-toxic substances are needed for postharvest preservation to efficiently extend the shelf life of leaves.

Reactive oxygen species (ROS) are generated as the by-products of plant aerobic respiration, which regulate various plant physiological processes under normal conditions [7]. However, excessive ROS may lead to oxidative damage under stress, including membrane lipid peroxidation (MDA), protein denaturation, and carbon metabolism perturbations [8]. Moreover, ROS dysregulation can accelerate senescence, negatively impacting the storage life of postharvest horticultural products, such as pak choi [9], pitaya [10], and banana [11]. Our recent findings indicated that ROS levels increase during storage, thus triggering mitochondrial oxidative damage of Chinese flowering cabbage [6]. Enzymatic systems (peroxidase (POD), ascorbate peroxidase (APX), glutathione reductase (GR), and catalase (CAT)) and non-enzymatic antioxidants (flavonoid, phenolic compounds, ascorbic acid (AsA), and glutathione (GSH)) counteract stress-induced ROS in plants. Therefore, these natural ROS-regulating mechanisms can provide valuable strategies for developing new postharvest preservation technologies for delaying leaf senescence.

The application of L-cysteine (L-Cys), a low-priced, natural, and eco-friendly reagent, can enhance antioxidant defense in plants. L-Cys, a sulfur-containing amino acid, is a key precursor for the synthesis of various sulfur-containing biomolecules, such as GSH, hydrogen sulfide (H_2_S), vitamins, and essential cofactors [12], which are vital for various metabolic and homeostatic processes in plants. Recent studies showed that L-cys can alleviate browning and microbial proliferation in fresh-sliced lotus root [13], thus increasing the phenol and flavonoid content in flat peach [14] and inhibiting leaf yellowing and respiration rates in parsley and peppermint leaves [15]. Additionally, L-cys directly scavenges ROS by reducing the sulfhydryl group (-SH) while also maintaining ROS balance in plants by modulating the antioxidant system. Recent studies have shown that L-cys treatment suppresses lipid peroxidation and enhances the antioxidant activity of superoxide dismutase (SOD), POD, and CAT enzymes in fresh-cut apples [16]. Also, L-cys treatment activates the ascorbate-glutathione (AsA-GSH) cycle, thus mitigating the brown rot of postharvest plums [17]. Furthermore, Huo et al. (2018) elucidated the regulatory mechanism of H_2_S during postharvest storage of fruits and vegetables and emphasized the crucial association between L-cys and endogenous H_2_S metabolism [18]. Specifically, in fresh mushroom slices, L-cys treatment can maintain high quality through stimulating endogenous H_2_S production [19]. Nonetheless, the role of L-cys in regulating leaf senescence in cabbage has not been systematically investigated.

In this study, cabbage leaves were treated with various concentrations of L-cys to explore its effects on chlorophyll degradation, ROS levels, antioxidant capacity, and endogenous H_2_S content. Therefore, this study may provide a solid foundation for a detailed investigation into the mechanistic effects of L-cys treatment on leaf senescence.

## 2. Materials and Methods

### 2.1. Plant Material and Treatment

The Chinese flowering cabbages used in this experiment were obtained from a large wholesale fruit and vegetable market in Guangzhou, Guangdong Province. After harvesting, the cabbages were pre-cooled and immediately delivered to the laboratory. The L-cys reagent used in this experiment was analytically pure and purchased from Sangon Biotech Co., Ltd., Shanghai, China.

Preliminary studies were conducted to determine the impact of varying L-cys concentrations on cabbage. The cabbages were immersed in L-cys solution for 3 min at concentrations of 0.25, 0.5, 0.75, and 1.0 g L^−1^. The control was treated similarly but immersed in ddH_2_O. All cabbages were dried in a cool area after treatment; cabbages subjected to the same treatment were transferred into the corresponding plastic baskets (15 per basket) and wrapped in polyethylene plastic bags to maintain moisture. To model a realistic commercial environment, the packaged cabbages were stored at 20 ± 0.5 °C with 80% relative humidity. During storage, the second set of leaves from the base of each plant were collected at each sampling point (0, 1, 3, 5, and 7 days). The collected leaves were grouped according to each time point and treatment. To ensure completely random sampling, the leaf tissues from each group were crushed into fragments under liquid nitrogen, homogenized, and stored at −80°C. Prior to analysis, a random aliquot was selected and ground into a fine powder for subsequent experimental measurements.

### 2.2. Determination of Chlorophyll Contents and Chlorophyll Fluorescence

After extraction and quantification of the chlorophyll content, imaging of the chlorophyll fluorescence and measurement of the maximum PSII quantum yield (variable fluorescence/maximal fluorescence, *Fv*/*Fm*) were performed following previous descriptions [2]. *Fv*/*Fm* was measured non-invasively using an Imaging-PAM-M series chlorophyll fluorometer (Heinz Walz GmbH, Effeltrich, Germany), which is equipped with a CCD camera for capturing digital images.

### 2.3. Determination of Nutritional Quality Parameters

The contents of nutritional quality were detected using spectrophotometric methods, following the instructions provided with the corresponding commercial assay kits of COMIN biotech Co., Ltd. (Suzhou, China). In this study, 0.2 g of frozen leaf tissue powder from cabbage samples subjected to different treatments was used for quantification. The results are expressed in the following units: total phenols content, soluble sugar, vitamin C content, protein content, and total flavonoid are reported in mg g^−1^ fresh weight (FW); amino acid content is presented in μg g^−1^ FW. The absorbance was measured using the Multiskan™ Sky Microplate Spectrophotometer (Thermo Fisher Scientific Inc., Waltham, MA, USA).

### 2.4. Determination of Parameters Associated with Oxidative Stress and Antioxidant Defense

MDA reacts with thiobarbituric acid (TBA), and their products exhibit peak absorption at 532 nm [20]. The frozen powder of cabbage leaf tissue was mixed in 1 mL of 5% trichloroacetic acid. The supernatant (0.2 mL) was taken and mixed with 0.6 mL of 0.67% TBA in water at 95 °C for half an hour. Subsequently, the tubes were cooled for centrifugation. MDA content was counted based on the absorbance of 532 nm and 600 nm. The data unit is expressed as nmol g^−1^ FW.

The analysis of H_2_O_2_ content and O_2_^•−^ production rate were tested by spectrophotometry, and according to the instruction of the corresponding kits (Suzhou COMIN biotechnology Co., Ltd.), the data units are expressed as µmol g^−1^ FW for H_2_O_2_ content and nmol g^−1^ min^−1^ FW for O_2_^•−^ production rate.

The total antioxidant capacity was determined using the 2,2-diphenyl-1-(2,4,6-trinitrophenyl) hydrazyl stable free radical (DPPH) assays [21]. Decolorization occurs when antioxidants are added to DPPH solutions, so the antioxidant capacity of antioxidants can be quantified using changes in absorbance and Trolox as a control system. The data unit is expressed as μmol Trolox g^−1.^ FW.

The analysis of •OH scavenging rate, O_2_^•−^ scavenging rate were measured using spectrophotometric methods, according to the instructions provided with commercial assay kits (Suzhou COMIN biotechnology Co., Ltd.); the data units are all expressed as percent (%).

### 2.5. Determination of Components in AsA-GSH Cycle

The contents of AsA, dehydroascorbate (DHA), GSH, and oxidized glutathione (GSSG) were determined following the analysis kits supplied by COMIN (Suzhou, China) using spectrophotometric methods; the data units are all expressed as mg g^−1^ FW.

APX, GR, dehydroascorbate reductase (DHAR), and monodehydroascorbate reductase (MDHAR) are crucial enzymes in the ASA-GSH cycle, which helps regenerate antioxidants and maintain cellular redox balance. Their activities were measured using spectrophotometric methods, following the protocols outlined in the analysis kits supplied by COMIN (Suzhou, China). One unit (U) of APX activity was defined as the amount of enzyme needed to oxidize 1 nmol AsA per minute. One unit of GR activity was considered as the enzyme required to oxidize 1 nmol NADPH per minute at PH 8.0. One unit of MDHAR activity was defined as the amount of enzyme for oxidizing 1 nmol NADH per minute. One unit of DHAR activity was the enzyme required to produce 1 nmol AsA per minute. The data unit of all the above-mentioned enzyme activity is expressed as U mg^−1^ FW.

### 2.6. Determination of Antioxidant Enzyme Activity

SOD, POD, and CAT are key antioxidant enzymes that protect cells from oxidative damage by detoxifying ROS. Their activities were measured using spectrophotometric methods, according to the procedures specified in the commercial testing products from COMIN (Suzhou, China). One unit (U) of POD activity was defined as the amount of enzyme required to produce a 0.005 absorbance change per second. For SOD activity, one unit (U) was defined as the amount of enzyme required to inhibit 50% of the nitroblue tetrazolium (NBT) reduction. The data unit of the above-mentioned enzyme activity is expressed as U mg^−1^ FW.

The determination of CAT activity followed a similar methodology as described by [22], with some modifications. This was based on the rate of decomposition of H_2_O_2_. H_2_O_2_ exhibits a distinctive absorption peak at 240 nm. The catalytic degradation of 1 nmol of H_2_O_2_ per minute was defined as one U of CAT activity; the data unit is expressed as U mg^−1^ FW.

### 2.7. Determination of Endogenous H_2_S Content and L-Cysteine Desulfhydrase (LCD) Activity

The endogenous H_2_S content was measured based on the method of Hu et al. [23]. The H_2_S content was measured using a reaction with acetic acid zinc, N, N-dimethyl P-phenylenediamine, and ferric ammonium sulfate, which forms a methylene blue with a maximum absorption peak at 665 nm. Based on the absorption value, the H_2_S concentration was determined and expressed as nmol g^−1^ FW.

Measurement of LCD activity was performed as previously depicted by Li [24], with some modifications. First, 0.2 g of leaf tissues was weighed and grounded into powder under liquid nitrogen. Subsequently, 1 mL of Tris-HCl buffer (20 mM, pH 8.0) was added to dissolve the powder into a suspension. After sufficient extraction, the mixture was centrifuged to collect the supernatant. The assay mixture (1 mL total volume) contained 0.8 mM L-cysteine, 2.5 mM dithiothreitol (DTT), 100 mM Tris-HCl (Ph 9.0), and 10 μg of supernatant. After a 15 min incubation at 37 °C, the reaction was terminated, and the amount of H_2_S produced was quantified using the methylene blue method previously described. One unit (U) of LCD activity was defined as the amount of enzyme required to produce 1 nmol of H_2_S per minute. The data unit is expressed as U mg^−1^ FW.

### 2.8. RNA Extraction and Real-Time Quantitative PCR (RT-qPCR)

The extraction of total RNA from flowering cabbage leaves and PCR reaction was conducted in accordance with the previously described methodology [6]. The primer sequences were designed in primer 3 and are shown in Table S1. *BrActin* (AF111812) was selected as the internal reference gene.

### 2.9. Statistics Analysis

All experiments followed a completely randomized design. Data were obtained from three independent biological replicates and are expressed as the mean ± SE. The differences between groups on days 0, 1, 3, 5, and 7 were assessed for statistical significance using Student’s *t*-test. *p* < 0.05 was considered significant, and *p* < 0.01 was considered extremely significant. GraphPad Prism 10.4 software (Graph Pad Software, San Diego, CA, USA) was used to perform all analyses.

## 3. Results

### 3.1. Impact of L-Cysteine Treatment on Color Changes and Chlorophyll Degradation

Leaf yellowing is a key sign of cabbage senescence and a reliable marker for assessing its progression. In this study, the second leaf from the bottom was consistently observed on day 5 to evaluate the effect of varying L-cys concentrations on leaf senescence. The results showed that leaves in the control were completely yellow at 5 d (Figure S1). Furthermore, 0.25 g L^−1^ L-cys did not significantly delay leaf yellowing, while the 0.5 g L^−1^ L-cys treatment effectively preserved the green color of cabbage. In contrast, 0.75 g L^−1^ and 1.0 g L^−1^ L-cys exacerbated the yellowing phenotype. As a result, a concentration of 0.5 g L^−1^ L-cys was selected as the optimal concentration for regulating senescence in cabbage leaves.

**Figure 1:**
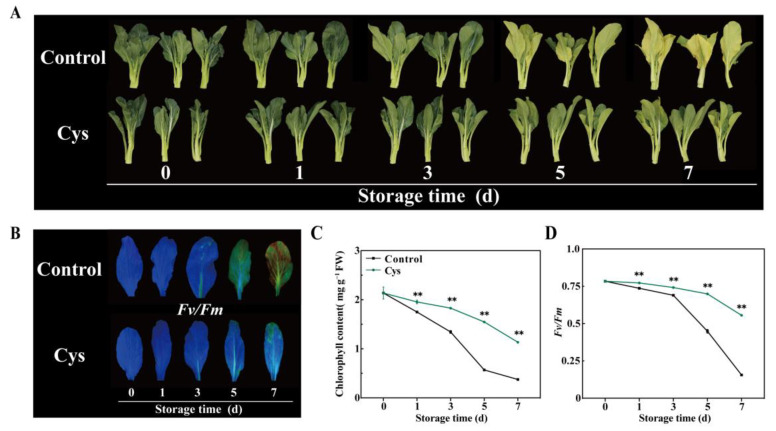
Changes in visual appearance of cabbage plants (**A**), chlorophyll fluorescence (**B**), chlorophyll content (**C**), and *Fv*/*Fm* ratio (**D**) of cabbage leaves treated with L-cys during leaf senescence. Error bars with data points represent the mean ± S.E. ** denotes a significant difference between control and L-cys treatment leaves at *p* < 0.01.

The effect of 0.5 g L^−1^ L-cys was assessed over the whole storage period. The leaves in the control group exhibited rapid yellowing by day 3, while L-cys-treated leaves maintained the green color, with only slight yellowing observed at the termination of the storage. Furthermore, fluorescence in the control group shifted from blue to green by day 3 and to brown by day 7 (Figure 1B). In contrast, the fluorescence of L-cys-treated leaves remained relatively stable, with minimal color change by days 5 and 7, reflecting delayed chlorophyll degradation. In addition, chlorophyll content (Figure 1C) and fluorescence *Fv*/*Fm* ratio (Figure 1D) declined in both groups. However, the reduction was significantly slower in the L-cys-treated leaves than in the control. For example, chlorophyll content in the control leaves and L-cys-treated leaves decreased by 82.45% and 46.9%, respectively (day 7). Similarly, the fluorescence *Fv*/*Fm* ratio in the control leaves and L-cys-treated leaves decreased by 80.13% and 29.1%, respectively. Gene expression analysis (Figure S2) revealed that the expression of genes associated with chlorophyll breakdown (*BrNYC1*, *BrNOL*, *BrPPH*, *BrPAO*, *BrNYE*, *BrSGR1,* and *BrSGR2*) in the L-cys-treated leaves was reduced by 5.95-, 3.68-, 1.35-, 3.80-, 3.02-, and 1.54-fold, respectively, compared with the control (on day 7). Additionally, L-cys treatment significantly downregulated the senescence marker gene *BrSAG12* by about 11-fold compared with control leaves (on day 7). These results suggest that L-cys treatment can effectively mitigate chlorophyll degradation and delay leaf senescence in cabbage.

### 3.2. Impact of L-Cysteine Treatment on Nutritional Quality

**Figure 2:**
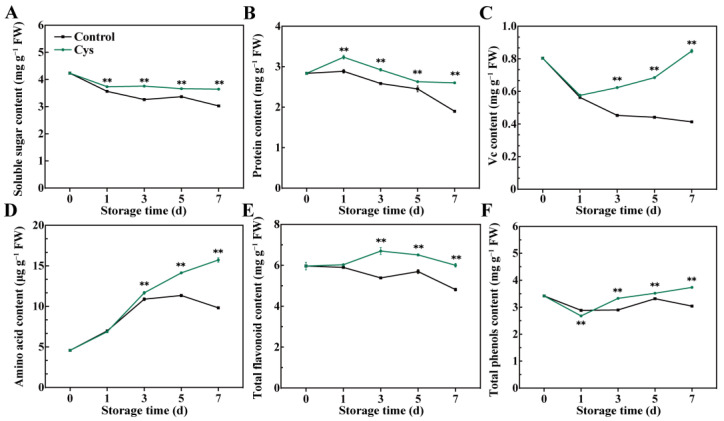
Changes in nutritional quality in Chinese flowering cabbage leaves treated with L-cys during leaf senescence, including contents of soluble sugar (**A**), protein (**B**), Vitamin C (**C**), amino acid (**D**), total flavonoids (**E**), and total phenols (**F**). Error bars with datapoints represent the mean ± S.E. ** denotes a significant difference between control and L-cys treatment leaves at *p* < 0.01.

Key nutrient components were measured during the storage period to evaluate the influence of L-cys treatment on cabbage’s nutritional properties. Notably, soluble sugar and protein content decreased over time in both control and L-cys-treated leaves (Figure 2A,B). However, L-cys treatment significantly slowed these reductions. Compared with day 0, the soluble sugar content in control sample and L-cys-treated sample decreased by 28.5% and 13.9%, respectively (day 7). Interestingly, vitamin C content (Figure 2C) decreased by 48.6% in control leaves and rose by 5.46% in L-cys-treated leaves (from day 0 to day 7). Furthermore, amino acid content increased in both groups, peaking on day 7 (Figure 2D). Specifically, the amino acid content in the L-cys-treated group and control group reached 15.72 μg g^−1^ FW and 9.82 μg g^−1^ FW, respectively (on day 7), reflecting a 243% and 114.5% increase, respectively (compared with day 0). Total flavonoid and total phenol contents showed distinct fluctuations with storage time (Figure 2E,F). The flavonoid content in L-cys leaves first increased (peaking on day 3), then decreased. Notably, flavonoid content was notably higher in the L-cys leaves than in the control leaves after 3 d. Similarly, total phenol content in L-cys leaves slightly fluctuated, showing an overall increasing trend during storage. Moreover, the L-cys-treated leaves exhibited a notably higher total phenol content compared to the control leaves except for day 1. The findings suggest that L-cys application helps retain the nutritional value of cabbage leaves throughout storage.

### 3.3. Effect of L-Cysteine Treatment on ROS Metabolism During Cabbage Leaf Senescence

**Figure 3:**
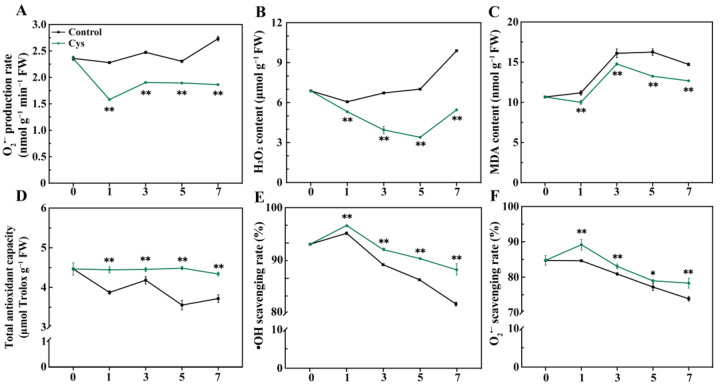
Changes in O_2_^•−^ production rate (**A**), H_2_O_2_ content (**B**), MDA content (**C**), total antioxidant capacity (**D**), •OH scavenging rate (**E**), and O_2_^•−^ scavenging rate (**F**) in Chinese flowering cabbage leaves treated with L-cys during the leaf senescence. Error bars with data points represent the mean ± S.E. * and ** denote a significant difference between control and L-cys treatment leaves at *p* < 0.05 and *p* < 0.01, respectively.

The ROS-induced oxidative injury to postharvest cabbage is significantly associated with leaf senescence. Herein, the O_2_^•−^ production rate, H_2_O_2_, and MDA content, as indicators of ROS accumulation, increased in both groups (Figure 3A–C). The O_2_^•−^ production rate of the control sample increased by 15.76% on day 7 (from 2.36 to 2.73 nmol g^−1^ min^−1^ FW) (Figure 3A). In contrast, L-cys treatment decreased the O_2_^•−^ production by 21.01% compared with day 0 (from 2.36 to 1.86 nmol g^−1^ min^−1^ FW). Similarly, the H_2_O_2_ content in the control sample increased throughout storage duration, while the H_2_O_2_ content in leaves treated by L-cys treatment first decreased and then increased after day 5 (Figure 3B). Notably, H_2_O_2_ content was significantly lower in the L-cys sample (44.88% lower) compared to the control by the end of the storage period. Additionally, the MDA content in the control sample increased by 38.06% from day 0 to day 7 (14.72 nmol g^−1^ FW). In contrast, the MDA content in the L-cys-treated sample increased by 18.87% (12.68 nmol g^−1^ FW) (Figure 3C).

ROS plays a crucial role in mitigating oxidative damage due to its scavenging capacity. In this study, the total antioxidant capacity of the L-cys-treated sample remained stable, with a minor reduction of only 2.82% from day 0 to day 7, maintaining a value of 4.4 μmol Trolox g^−1^ FW (Figure 3D). In contrast, the total antioxidant capacity of the control sample significantly decreased by 16.73% (3.71 μmol Trolox g^−1^ FW) compared with day 0. The scavenging rates of O_2_^•−^ and •OH showed similar trends (Figure 3E,F), first increasing and then decreasing (peaking on day 1). Notably, the O_2_^•−^ and •OH scavenging rates were significantly higher in the sample treated with L-cys.

### 3.4. Effect of L-Cysteine Treatment on the Modulation of AsA-GSH Cycle-Associated Antioxidant Levels

AsA content in both groups is shown in Figure S3A. AsA content in the control group continuously decreased (by 48.6%), reaching 0.41 mg g^−1^ FW on day 7 compared with day 0). In contrast, AsA content in the L-cys-treated group first decreased (day 1) and then increased, reaching 0.85 mg g^−1^ FW by day 7. AsA can oxidize to DHA. The DHA content is shown in Figure S3B. DHA content increased in both groups. Interestingly, the DHA content in the control leaves rapidly increased by 940% during the whole storage period. The DHA content in the L-cys-treated group increased by 178.95% from day 0 to day 7. The AsA/DHA ratio decreased in both groups during the storage (Figure S3C). However, the AsA/DHA ratio in the leaves treated by L-cys was much greater than in the control.

GSH and GSSG content (Figure S3D,E) showed more complex trends. For instance, GSH content increased in both groups, rapidly increasing in the treated leaves. Moreover, the GSH content in control leaves and the treated leaves increased by 60.27% and 101.4%, respectively (from day 0 to 7). Furthermore, the GSSG content showed an overall increase during the whole storage time. However, GSSG levels were significantly lower in leaves treated by L-cys (17.5 mg g^−1^ FW) than in the control (23.3 mg g^−1^ FW) at day 7. The GSH/GSSG ratio increased in both groups, especially in the leaves treated by L-cys, particularly between days 0 and 3 (Figure S3F).

### 3.5. Effect of L-Cysteine Treatment on the Activity and the Expression of AsA-GSH Pathway-Related Enzymes

**Figure 4:**
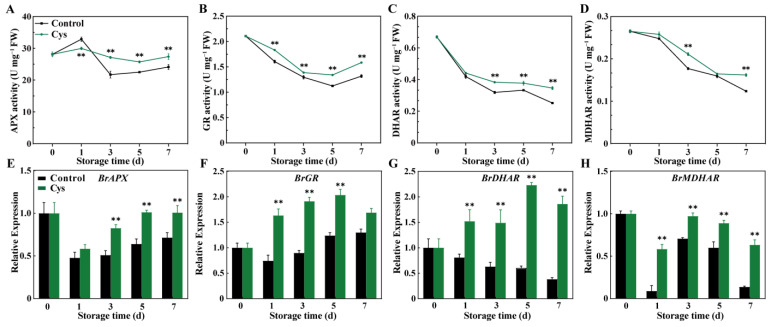
Changes in APX activity (**A**), GR activity (**B**), DHAR activity (**C**), and MDHAR activity (**D**) as well as relative expression levels of *BrAPX* (**E**), *BrGR* (**F**), *BrDHAR* (**G**), and *BrMDHAR* (**H**) in Chinese flowering cabbage leaves treated with L-cys during storage. Error bars with data points represent the mean ± S.E. ** denotes a significant difference between control and L-cys treatment leaves at *p* < 0.01.

L-cys treatment effectively enhanced antioxidant enzyme activities and gene expression in the AsA-GSH cycle during cabbage leaf storage. Enzyme activity analysis (Figure 4A,B) showed that APX and GR activities in the control sample first significantly increased and then rapidly declined. In contrast, APX and GR activities in the leaves treated by L-cys were relatively stable. Specifically, APX activity in L-cys-treated sample and control sample decreased by 2.5% and 14.12%, respectively. GR activity in the L-cys-treated sample (1.58 U g^−1^ FW) surpassed that in the control group (1.37 U g^−1^ FW) on day 7. Moreover, DHAR and MDHAR activities decreased in both groups, but the decline was slower in the L-cys-treated sample (Figure 4C,D). DHAR and MDHAR levels in the L-cys-treated group were enhanced by 37.5% and 30.9% in comparison to control sample at day 7, respectively.

Gene expression analysis (Figure 4E–H) revealed that L-cys treatment up-regulated *BrAPX*, *BrGR*, *BrDHAR*, and *BrMDHAR*. Specifically, *BrAPX* and *BrGR* expression were elevated with the L-cys treatment throughout the storage compared to the control. Notably, with the L-cys treatment, *BrDHAR* expression increased by 61% by day 7, while it decreased by 85.9% in the control group. *BrDHAR* expression in sample treated with L-cys was 484% higher compared to the control (day 7). Similarly, *BrMDHAR* expression in the L-cys-treated sample was 661% higher on day 1 and 465% higher on day 7 compared with the control.

### 3.6. Effects of L-Cysteine Treatment on the Activities and Gene Expression of POD, SOD, and CAT

**Figure 5:**
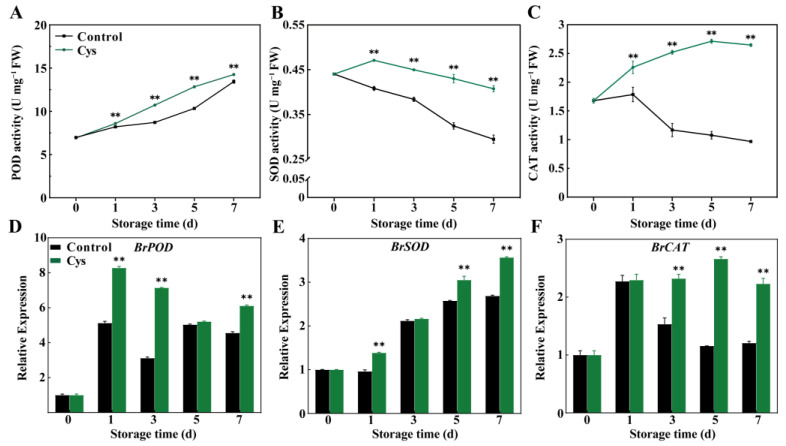
Changes in POD activity (**A**), SOD activity (**B**), and CAT activity (**C**) as well as relative expression levels of *BrPOD* (**D**), *BrSOD* (**E**), and *BrCAT* (**F**) in Chinese flowering cabbage leaves treated with L-cys during storage. Error bars with data points represent the mean ± S.E. ** denotes a significant difference between control and L-cys treatment leaves at *p* < 0.01.

L-cys treatment significantly modulated antioxidant enzyme activities as well as gene expression during storage. POD activity exhibited an overall increasing trend in both groups (Figure 5A). POD activity in the L-cys-treated leaves and in control increased by 106% (from 6.92 to 14.26 U mg^−1^ FW) and 92.5%, respectively. Furthermore, SOD activity (Figure 5B) declined in both groups, especially in the control. Interestingly, CAT activity in the control group decreased by 42.35% and increased by 57.6% in the L-cys-treated group, reaching 2.65 U mg^−1^ FW on day 7 (Figure 5C). Gene expression analysis (Figure 5D–F) revealed that the expression of *BrPOD*, *BrSOD*, and *BrCAT* slightly fluctuated in the two groups. Compared to the control, the transcript accumulation of *BrPOD*, *BrSOD*, and *BrCAT* was elevated following L-cys treatment.

### 3.7. Effect of L-Cys Treatment on the Endogenous H_2_S Levels, LCD Activity, and Gene Expression of BrLCD, BrCYS-C1, BrCYS-D1

**Figure 6:**
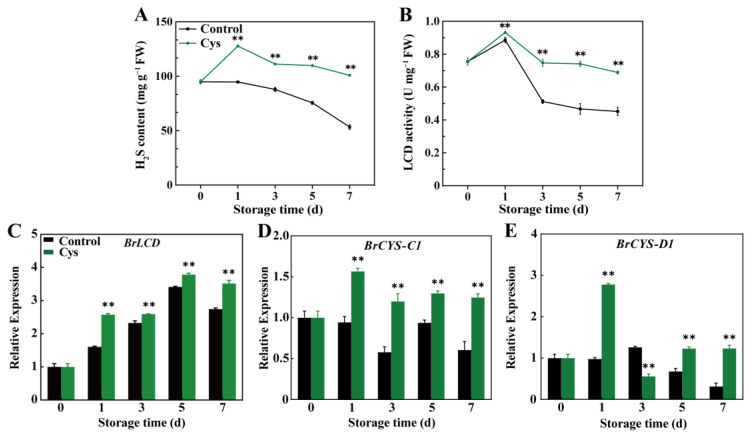
Changes in endogenous H_2_S content (**A**) and LCD activity (**B**) as well as relative expression levels of *BrLCD* (**C**), *BrCYSC1* (**D**), and *BrCYSD1* (**E**) in Chinese flowering cabbage leaves treated with L-cys during leaf senescence. Error bars with data points represent the mean ± S.E. ** denotes a significant difference between control and L-cys treatment leaves at *p* < 0.01.

H_2_S content significantly increased with the L-cys treatment from day 0 to day 1 and then gradually decreased, reaching 100.9 nmol g^−1^ FW on day 7, representing a 6.3% increase compared to day 0 (Figure 6A). In contrast, H_2_S content in the control group decreased by 40.2%, reaching 53.2 nmol g^−1^ FW by day 7. H_2_S content in the L-cys treatment was higher by 34.85%, 26.53%, 45.36%, and 89.17% than in the control during storage, respectively. LCD activity (Figure 6B) showed the same trend in both groups, peaking on day 1 and then decreasing. LCD activity in the control group decreased by 40.17% on day 7 compared with day 0 (0.45 U mg^−1^ FW). However, LCD activity slightly decreased with the L-cys treatment compared with the control (8.75%, 0.69 U mg^−1^ FW).

Gene expression analysis (Figure 6C–E) demonstrated that L-cys treatment increased the relative expression of *BrLCD*, *BrCYS-C1*, and *BrCYS-D1*. Furthermore, *BrLCD* expression was consistently higher in the L-cys-treated group than in the control group (from days 0 to 7) by about 1.6–1.28 times (Figure 6C). The expression of *BrCYS-C1* (Figure 6D) in control leaves and L-cys-treated leaves decreased by 85% and 61.5%, respectively (on day 7). Similarly, *BrCYS-D1* expression (Figure 6E) in the L-cys-treated group was 391% higher (1.23 times) than in the control group (day 7).

### 3.8. Correlation Analysis of Chlorophyll Degradation, Nutritional Quality, ROS Metabolism, and H_2_S Metabolism

**Figure 7:**
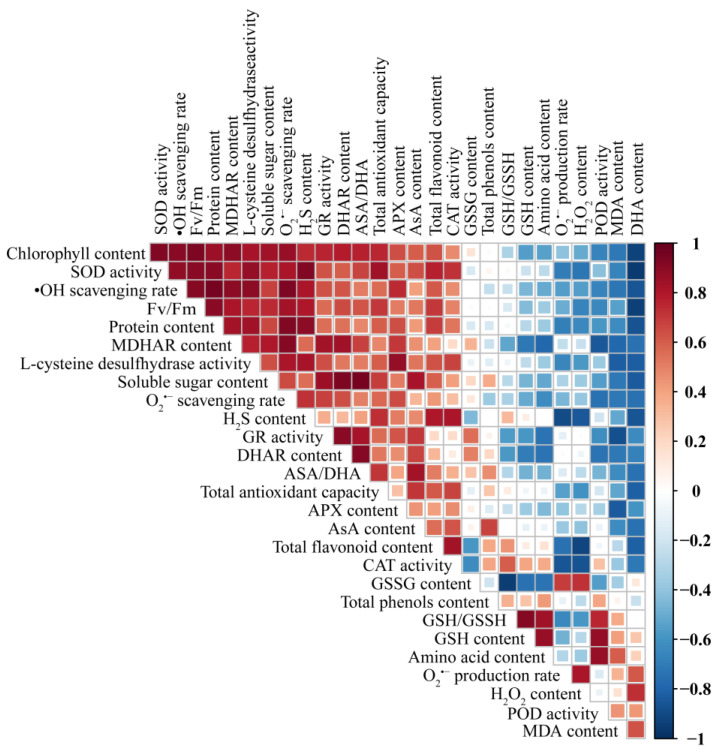
Pearson correlation analysis of the effect of L-cys treatment on postharvest Chinese flowering cabbages. The color red is used to represent a positive correlation, whereas the color blue is used to represent a negative correlation.

The relationships among chlorophyll degradation, nutritional quality, ROS metabolism, and H_2_S metabolism in L-cys-treated and control groups were assessed using Pearson correlation analysis (Figure 7). MDA was negatively correlated with the antioxidant substances of AsA-GSH pathway (AsA and GSH) and enzymatic antioxidants (POD, SOD, and CAT). Conversely, MDA was positively correlated with H_2_O_2_ content, O_2_^•−^ production, and •OH formation, indicating increased oxidative stress. Furthermore, the endogenous H_2_S content was positively correlated with several critical parameters associated with oxidative stress regulation. Specifically, endogenous H_2_S content was positively correlated with LCD enzyme, antioxidant capacity, key antioxidant enzymes (POD, SOD, and CAT), and enzymes in the AsA-GSH pathway (APX, GR, DHAR, and MDHAR).

## 4. Discussion

L-cys is a natural amino acid. As a food additive, it safely used in the food industry [25]. In the present study, 0.5 g L^−1^ L-cys maintained the Chinese flowering cabbage green during storage. Compared with the control leaves, the 0.5 g L^−1^ L-cys treatment was associated with a slower rate of chlorophyll loss and a lesser decline in *Fv*/*Fm* values (Figure 1A–D). Recent experiments suggested that 0.5 g L^−1^ L-cys inhibited relative expression of the catabolic genes involved in chlorophyll degradation, including *BrPAO*, *BrPPH*, *BrNYC1*, *BrNYE1*, and *BrNOL* [26,27,28], thus alleviating the cabbage leaf yellowing. Similarly, the present study shows that L-cys treatment significantly downregulated key chlorophyll catabolic genes (*BrNYC1*, *BrNOL*, *BrPPH*, *BrPAO*, *BrNYE*, *BrSGR1*, and *BrSGR2*) and senescence marker gene (*BrSAG12*) (Figure S2A–H). These findings suggest that 0.5 g L^−1^ L-cys treatment can maintain a higher chlorophyll content and green phenotype by inhibiting the chlorophyll degradation. Additionally, L-cys treatment preserved higher levels of soluble sugars, proteins, and vitamin C (Figure 2A–C), indicating that it can maintain the nutritional quality of the cabbage.

A multitude of investigations have indicated that exogenous L-cys is capable of efficiently modulating the enzymatic browning process in fresh-cut fruits and vegetables [29,30]. However, during the senescence of non-fresh-cut vegetables, disrupted ROS homeostasis during leaf senescence leads to accumulation of ROS such as O_2_^•−^ and H_2_O_2_, which constantly attack cell membrane lipids, impairing physiological and metabolic functions and accelerating leaf senescence [31]. In this study, the O_2_^•−^ production rate (Figure 3A), H_2_O_2_ levels, and MDA content were significantly increased (Figure 3B,C) during leaf senescence. However, L-cys treatment reduced the production rate of O_2_^•−^ and also inhibited the accumulation of H_2_O_2_ and MDA. Other studies have also revealed that L-cys reduces ROS accumulation in peach [14], tuberose cut flowers [32], and plums [17]. Herein, L-cys-treated leaves maintained a higher total antioxidant capacity and OH^•^ and O_2_^•−^ scavenging rate (Figure 3D–F), indicating that L-cys treatment alleviates ROS accumulation and its impact through ROS scavenging.

The AsA-GSH cycle is the key ROS scavenging system. AsA and GSH are crucial redox agent in this pathway, acting directly or in coordination with related associated enzymes (APX, MDHAR, DHAR, and GR) to detoxify H_2_O_2_ and maintain redox homeostasis [33]. APX reduces H_2_O_2_ to water because of the high redox potential of AsA, converting AsA into its oxidized form, DHA. AsA is then regenerated by cycling enzymes (MDHAR and DHAR). Meanwhile, GSH is oxidized to GSSG during ROS detoxification and reduced to GSH by GR [34]. Studies have suggested that enhancing the activity of these enzymes can promote the regeneration of AsA and GSH in the cycle, thus boosting oxidative stress resistance and delaying senescence in horticultural products [6,10]. For instance, previous research [9] reported that 1-MCP can delay the decrease in APX, GR, MDAR, and DHAR activity, leading to high AsA and GSH levels, effectively eliminating ROS and retarding the senescence of pak choi. Furthermore, 0.4% L-cys increases APX activity in flat peach, thus increasing AsA content, thereby scavenging H_2_O_2_ and protecting against ROS-induced oxidative damage [14]. In this study, the enzyme activities of APX, GR, DHAR, and MDHAR (Figure 4A–C), together with their expression levels (*BrAPX*, *BrGR*, *BrDHAR*, and *BrMDHAR*) (Figure 4 E–H), were significantly up-regulated in L-cys-treated leaves. The increased activity can promote the recycling of antioxidants, thus increasing AsA and GSH levels (Figure S3A,D) along with AsA/DHA and GSH/GSSG ratios (Figure S3C,F). Therefore, L-cys treatment may activate the antioxidant capacity by stimulating the AsA-GSH cycle, thus minimizing free radical damage.

SOD, CAT, and POD, as vital ROS-scavenging enzymes, are vital in mitigating oxidative stress. SOD facilitates the conversion of the O_2_^•−^ to H_2_O_2_, a less reactive compound. Meanwhile, CAT and POD convert H_2_O_2_ into H_2_O and oxygen [35,36]. In our prior research, diverse postharvest treatment modalities, such as the utilization of melatonin [2] and cytokinin [2,37], have been demonstrated to be efficacious in augmenting ROS-scavenging enzyme activity. This augmentation plays a crucial role in decelerating the leaf senescence of Chinese flowering cabbage and consequently prolonging its postharvest shelf life. Similarly, in this study, L-cys treatment activated the activity of SOD, CAT, and POD enzymes (Figure 5A–C) and up-regulated genes encoding these enzymes (Figure 5D–F), which is consistent with the observed decrease in ROS levels (Figure 3A–C), subsequently helping to delay leaf senescence in Chinese flowering cabbage. Other studies have also shown that L-cys can activate CAT and SOD enzymes in litchi fruit [38] and fresh-cut lotus root slices [13], reducing lipid peroxidation and delaying fruit senescence. A recent study suggested that higher gene expression of *MdSOD*, *MdCAT6*, and *MdCAT13* in L-cys-treated fresh-cut apples can improve antioxidant defense and inhibit browning [16]. These findings indicated that L-cys treatment scavenges ROS by activating antioxidant enzymatic and transcript levels of related genes, thus delaying cabbage leaf senescence.

L-cys is a key precursor in the biosynthesis of H_2_S, an endogenous gaseous transmitter involved in various physiological processes. A recent study has shown that H_2_S enhances stress tolerance, boosting antioxidant enzyme activity and delaying postharvest senescence in horticultural products [39]. For example, H_2_S pretreatment is a crucial antioxidative signaling molecule activating antioxidative enzymes such as *cAPX*, *CAT*, *GR*, and *MnSOD*, thus mitigating the impact of non-biological stress in strawberry [40]. In addition, H_2_S can regulate senescence, thus inhibiting the leaf yellowing of water spinach [23] and prolonging the longevity of cut flowers [41]. The use of L-cys as a precursor for H_2_S synthesis could have significant environmental and economic benefits, as it provides a natural and sustainable method to enhance postharvest quality, reducing reliance on chemical preservatives and minimizing waste, although its application may face challenges concerning controlling H_2_S production. However, direct application of H_2_S is challenging due to its pungent odor. As a result, the preservation effect can be achieved by stimulating endogenous H_2_S synthesis in postharvest horticulture products. For instance, H_2_S treatment enhances hawthorn cold resistance by increasing H_2_S production and boosting antioxidant activities [42]. Exogenous H_2_S fumigation postpones the postharvest senescence of broccoli in a dose-dependent fashion. Moreover, H_2_S sustains relatively elevated levels of chlorophyll, which implies the involvement of H_2_S in retarding the postharvest senescence process of broccoli [43]. Research has recently confirmed that L-cys improves the preservation period extension in fresh mushroom slices due to the activation of H_2_S production and associated signaling pathways [19]. In this study, endogenous H_2_S levels were significantly increased in the L-cys-treated sample (Figure 6A), suggesting that L-cys delays leaf senescence by regulating endogenous H_2_S biosynthesis. Additionally, L-cys significantly activated LCD activity, which is crucial for L-cys catabolism, thus producing H_2_S.

Additional desulfurizing enzymes, including isoenzymes of β-cyanoalanine synthase (β-CAS) such as CYS-C1 and CYS-D1, also promote H_2_S production [44]. These enzymes catalyze the conversion of L-cys and hydrogen cyanide into H_2_S and 3-cyano-L-alanine, leading to simultaneous cyanide detoxification and H_2_S production [45,46]. Herein, the relative expression of these L-cysteine-related desulfurizing enzyme genes was determined to identify the specific enzymes involved in this process. L-cys treatment up-regulated *BrLCD*, *BrCYS-C1*, and *BrCYS-D1* during the entire storage period compared with the control group (Figure 6C–E). Sun et al. also showed that NaHS treatment can increase *DcLCD* expression, triggering endogenous H_2_S biosynthesis [47]. Furthermore, 1 mM cysteine increases the expression of the *CYSC1* in Arabidopsis suspension cell cultures [48]. Overall, L-cys can stimulate the biosynthesis of endogenous H_2_S by activating the LCD activity and up-regulating *BrLCD*, *BrCYS-C1*, and *BrCYS-D1*.

## 5. Conclusions

Herein, results showed that 0.5 g L^−1^ L-cys treatment delayed chlorophyll degradation and leaf senescence in cabbage leaves at 20 °C. Furthermore, L-cys treatment reduced ROS and MDA levels, stimulated the AsA-GSH cycle, and maintained high levels of AsA and GSH. These effects were achieved through increased activity and expression of key antioxidant enzymes in this cycle, including GR, CAT, DHAR, and MDHAR. Additionally, L-cys-increased the activities of POD, SOD, and CAT as well as the transcript levels of related genes. These findings indicate that L-cys treatment can maintain redox homeostasis by promoting ROS scavenging and limiting ROS production. Furthermore, L-cys treatment increased endogenous H_2_S levels by up-regulating LCD activity and the expression of H_2_S biosynthesis-related genes (*BrLCD*, *BrCYS-C1*, and *BrCYS-D1*). Taken together, these findings indicate that L-cys can delay the onset of leaf senescence and improve the storage quality of Chinese flowering cabbage.

## Data Availability

The original contributions presented in the study are included in the article/Supplementary Materials, further inquiries can be directed to the corresponding author.

## Supplementary Materials

The following supporting information can be downloaded at: https://www.mdpi.com/article/10.3390/foods14010029/s1, Figure S1: Visual appearance of Chinese flowering cabbage leaves treated with various L-cys concentration at day 5 of storage at 20 ± 1 °C; Figure S2: Relative expression of the chlorophyll degradation genes *BrNYC1* (A), *BrNOL* (B), *BrPPH* (C), *BrPAO* (D), *BrNYE* (E), *BrSGR1* (F), and *BrSGR2* (G) and the senescence marker gene *BrSAG12* (H) during leaf senescence. Error bars with data points represent the mean ± S.E. ** denotes a significant difference between control and L-cys treatment leaves at *p* < 0.01; Figure S3: Changes in AsA (A), DHA (B), GSH (C), and GSSG (D) content as well as AsA/DHA (E) and GSH/GSSG (F) ratio content in Chinese flowering cabbage leaves treated with L-cys during leaf senescence. * and ** denote a significant difference between control and L-cys treatment leaves at *p* < 0.05 and *p* < 0.01, respectively; Table S1: Primer sequences used for qRT-PCR in the present study; Table S2: Correlation coefficients (r-values) and *p*-values of the Pearson correlation heatmap (Figure 7).
