# Supplementary material for: L-Cysteine Treatment Delays Leaf Senescence in Chinese Flowering Cabbage by Regulating ROS Metabolism and Stimulating Endogenous H2S Production
Source: Foods. 2024 Dec 25;14(1):29. doi: 10.3390/foods14010029 (PMC11719892; doi:10.3390/foods14010029)
Supplement: Supplementary file 1 [file foods-14-00029-s001.zip › Figure Legend.docx]

**Supplemental Figure S1:** Visual appearance of Chinese flowering cabbage leaves treated with various L-cys concentration at day 5 of storage at 20 ± 1 ^◦^C.

**Supplemental Figure S2:** Relative expression of chlorophyll degradation genes: *BrNYC1* (A)*, BrNOL* (B)*, BrPPH* (C)*, BrPAO* (D)*, BrNYE* (E)*, BrSGR1* (F)*, BrSGR2* (G) and senescence marker gene: *BrSAG12* (H) during leaf senescence. Error bars with data points represent the mean ± S.E. Symbol ** denote a significant difference between control and L-cys treatment leaves at P < 0.01.

**Supplemental Figure S3:** Changes in AsA (A), DHA (B), GSH (C) and GSSG (D) content, as well as AsA/DHA (E) and GSH/GSSG (F) ratio content in Chinese flowering cabbage leaves treated with L-cys during leaf senescence. Symbol*, ** denote a significant difference between control and L-cys treatment leaves at P < 0.05 and P < 0.01, respectively.

**Supplementary Table S1:** List of primers for qRT-PCR.

**Supplementary Table S2:** Correlation coefficients (r-values) and P-values of the Pearson correlation heatmap (Figure 7).
