# Supplementary figures and images for: L-Cysteine Treatment Delays Leaf Senescence in Chinese Flowering Cabbage by Regulating ROS Metabolism and Stimulating Endogenous H2S Production
Source: Foods. 2024 Dec 25;14(1):29. doi: 10.3390/foods14010029 (PMC11719892; doi:10.3390/foods14010029)

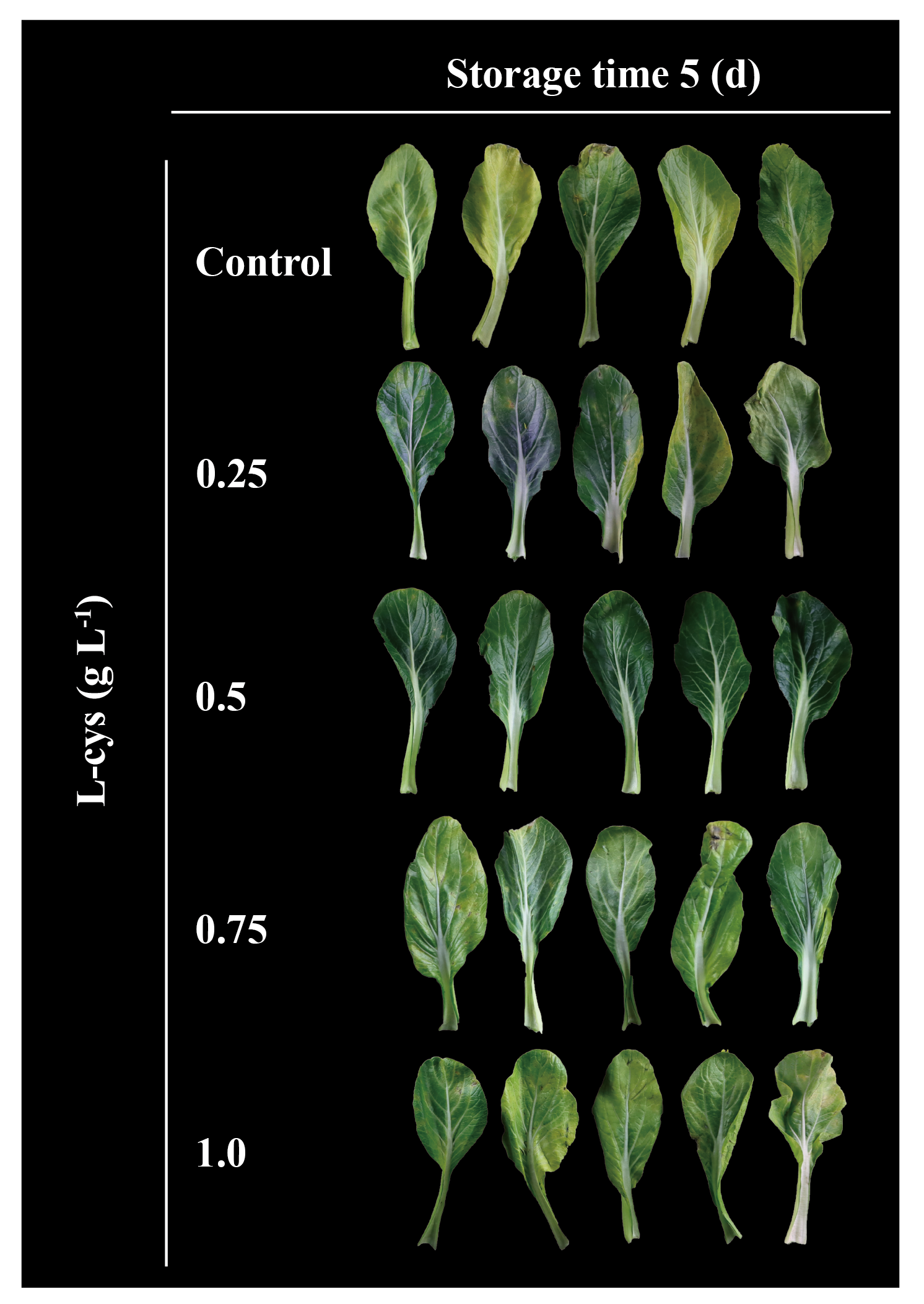

Supplement: Supplementary file 1 [file foods-14-00029-s001.zip › Fig.S1.tif]

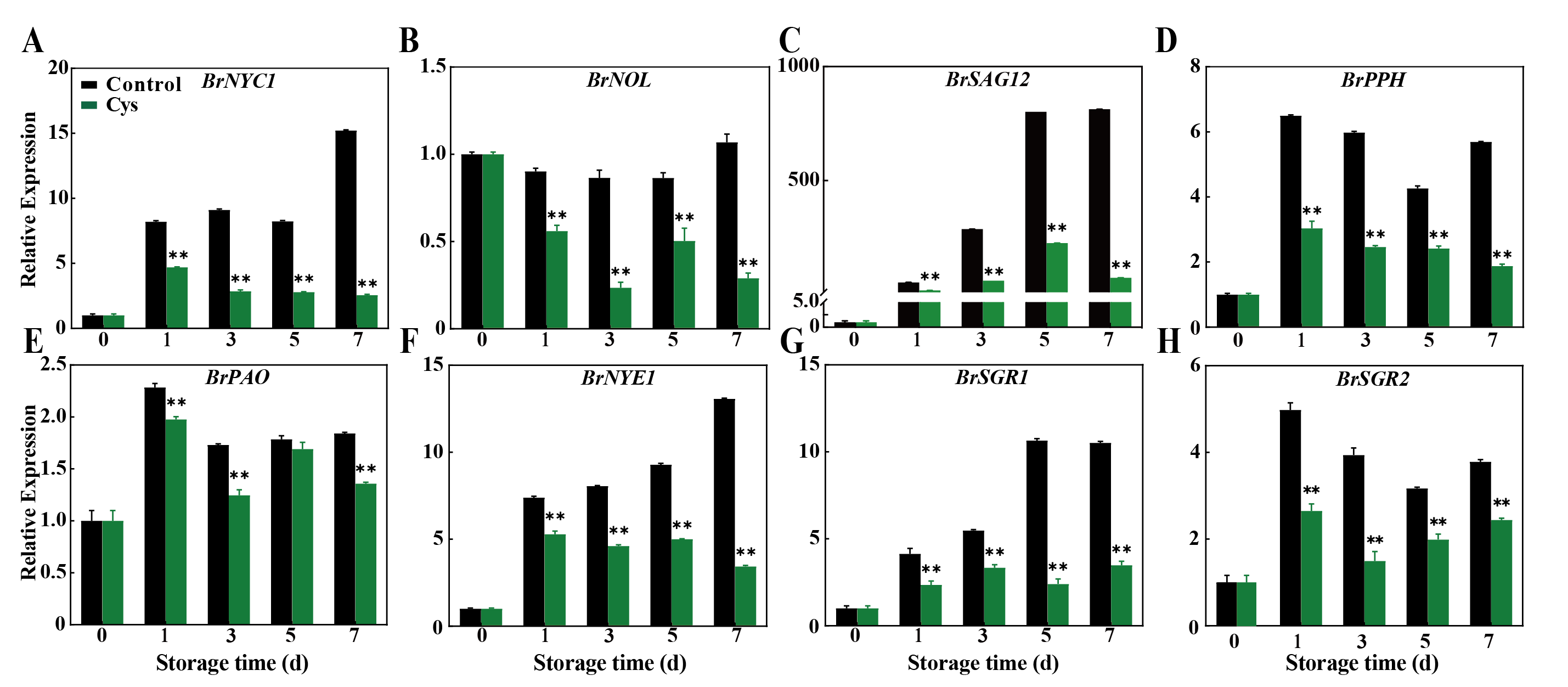

Supplement: Supplementary file 1 [file foods-14-00029-s001.zip › Fig.S2.tif]

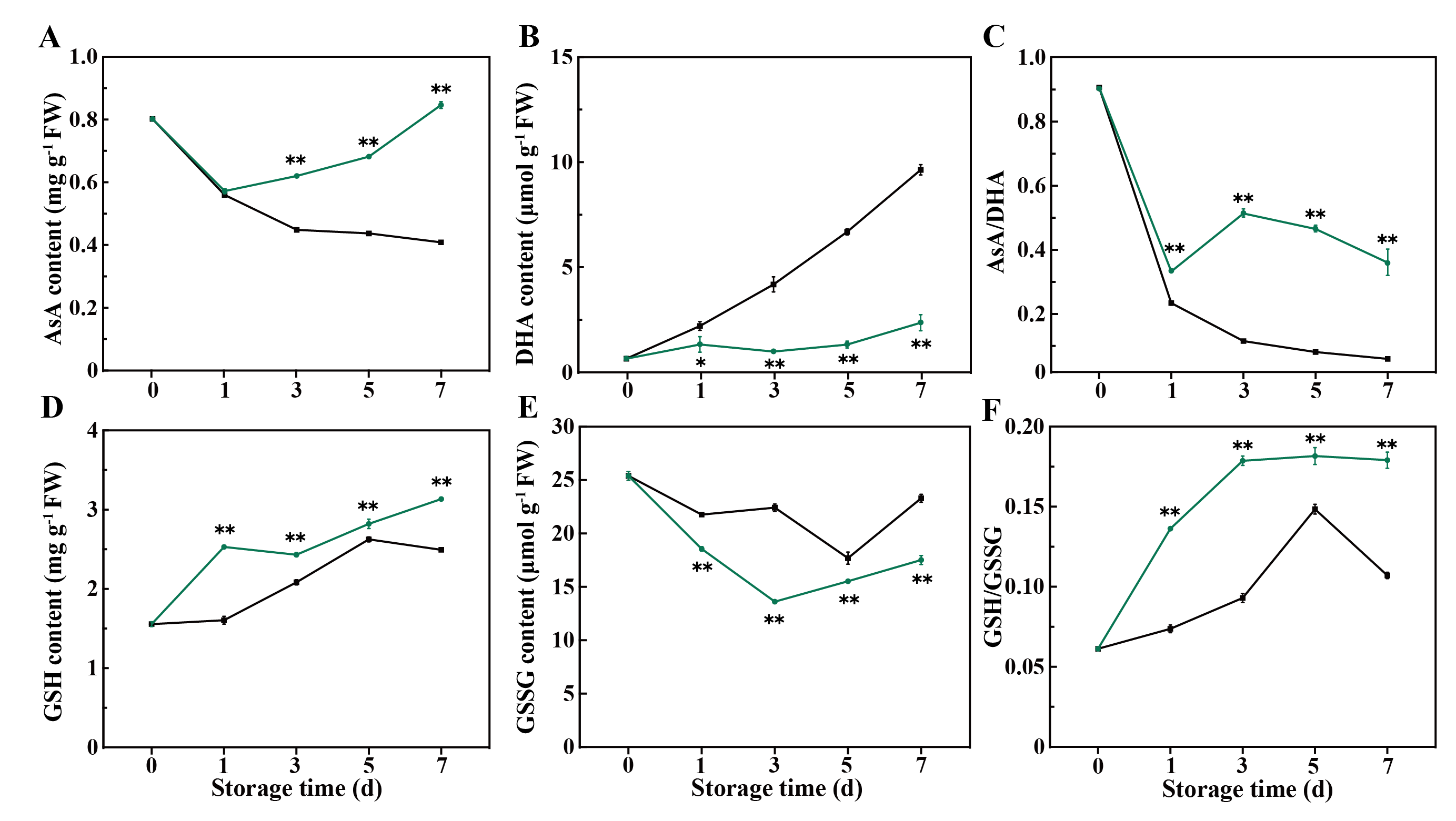

Supplement: Supplementary file 1 [file foods-14-00029-s001.zip › Fig.S3.tif]
